# Like soldiers on the front – a qualitative study understanding the frontline healthcare professionals’ experience of treating and caring for patients with COVID-19

**DOI:** 10.1186/s12913-021-06637-4

**Published:** 2021-07-07

**Authors:** Ilkay Dagyaran, Signe Stelling Risom, Selina Kikkenborg Berg, Ida Elisabeth Højskov, Malin Heiden, Camilla Bernild, Signe Westh Christensen, Malene Missel

**Affiliations:** 1 Department of Infectious Diseases, the Heart Center, Rigshospitalet, Copenhagen University Hospital, Copenhagen, Denmark; 2 Heart Center, Rigshospitalet, Copenhagen University Hospital, Copenhagen, Denmark; 3 Institute of Nursing and Nutrition, University College Copenhagen, Copenhagen, Denmark; 4 Department of Clinical Medicine, Faculty of Health and Medical Sciences, University of Copenhagen, Copenhagen, Denmark; 5 National Institute of Public Health, University of Southern Denmark, Odense, Denmark; 6 Department of Cardiothoracic Surgery, the Heart Center, Rigshospitalet, Copenhagen, Denmark

**Keywords:** 2019-nCoV; COVID-19, Coronavirus, Qualitative study, Ricoeur, Healthcare professionals, Work life balance

## Abstract

**Background:**

While people in the societies must stay home to reduce spread of the newly discovered coronavirus, healthcare professionals do the exact opposite. For them the coronavirus is an enemy that should be defeated as a part of one’s job. They do, however, also have a daily life with family while doing their work obligations. The purpose of this study was to gain an in-depth understanding of the frontline healthcare professionals’ experience of balancing work life and family life during the COVID-19 pandemic.

**Methods:**

A sample of 22 frontline healthcare professionals caring for patients with COVID-19 was included and interviewed individually from May to August 2020. Ricoeur’s phenomenological hermeneutical philosophy inspired the methodology in this study.

**Result:**

Frontline healthcare professionals treating and caring for patients with COVID-19 are, voluntarily or involuntarily, forced to be ready to change departments as well as being ready to face the unknown coronavirus. The frontline work leads to feelings of being abandoned among their families and friends due to the threat of bringing the infection home and spreading the virus. Although healthcare professionals are facing a working life filled with uncertainty and unpredictability impacting their family life, they express opposing feelings of being a part of something bigger.

**Conclusions:**

The work life balance for these healthcare professionals is threatened by changes in professional responsibilities, working hours and shifts. Fear of bringing the infection home challenges them ethically and creates a distance between healthcare professionals and their families, leading to a conflict within the individual if their work on the frontline is worth it - or if it is a too high price to pay. Despite facing a working life filled with uncertainty and unpredictability the healthcare professionals are being a part of something bigger that contributes to a fighting spirit and professional pride outweighing the negative consequences; like being soldiers on the front.

## Introduction

 Healthcare professionals (HCPs) worldwide have been praised for their frontline efforts in the care and treatment for patients with COVID-19 in the current pandemic situation. It has been reported how they approach these tasks with a stoic calm and an altruistic attitude as well as how their strong professional identity overrides most concerns about their own health. These HCPs see the coronavirus as an enemy that should be defeated as a part of one’s job. They do, however, also have a daily life with family and other interests while doing their work obligations. In this study we investigated how these frontline HCPs balance their work obligations and their family life during the current COVID-19 pandemic. The study offers an in-depth understanding of what is at stake for HCPs caring for and treating patients with COVID-19 infection while still maintaining a usual family life. These findings should be of interest to a broad worldwide readership within healthcare and will add knowledge to the growing COVID-19 evidence base and in developing supportive interventions targeted HCPs during pandemics in the future.

## Background

In January 2020 the World Health Organization declared a Public Health Emergency of International Concern as the newly discovered coronavirus, causing COVID-19 disease, was considered to have caused a global pandemic [1]. The COVID-19 disease mainly attacks the human respiratory system causing clinical symptoms such as coughing and dyspnea followed by fever and bilateral lung infiltrates shown by imaging [2, 3]. Individuals with severe symptoms and suspected infection are monitored and diagnosed in hospitals and are being isolated, while those with mild symptoms are isolated at home [4]. Studies show how this global pandemic pressures healthcare systems to the extreme worldwide with a pervasive workload for HCPs working against an unprecedented and contagious virus [5, 6]. To contain the potentially devasting consequences and limiting the spread of virus, many governments have imposed very strict and drastic measures in societies such as closure of unnecessary activities and recommendations of staying home [7]. While people in the societies must stay home and work from home to reduce spread of virus, HCPs do the exact opposite. HCPs are currently desperately needed and the world’s most important resource in the fight against the pandemic, but at the same time they represent one of the most vulnerable populations. Studies have documented how these HCPs are put under pressure both physically and psychologically due to increased workload, fear and anxiety of getting infected themselves and bringing the virus to other vulnerable patients or home to their family members [8–11]. The protection of HCPs is as such vital in continuing patient care in healthcare systems that are currently challenged by the pandemic, but also important in ensuring not spreading the virus.

Historically, nurses have always played an important role in prevention and control of infections and public health [12–14]. The fight against COVID-19 is, however, dependent on extraordinary efforts from, and collaboration between, various HCPs, e.g. nurses, doctors, physiotherapists, pharmacists and porters [15]. They are currently facing frontline care and treatments for patients with COVID-19. These HCPs have to do this job under new working conditions, both due to the physical work environment in newly created COVID-19 departments as well as professionally depending on their previous knowledge, skills and experiences. While research has described how HCPs during the first phase of the COVID-19 pandemic approached their obligations with a stoic and altruistic orientation towards their work [15], others have reported how questions of who will step up to the plate in this ongoing crisis arise [16]. These frontline HCPs are additionally forced to adapt to a job that can be unpredictable in relation to working hours and schedule which might impact and have consequences for their work life balance [17]. Work life balance refers to the ability of individuals to coordinate work and family obligations successfully which impact mental and physical health [18, 19], however, to our knowledge little attention has been paid to this aspect of the caring and treatment responsibilities for frontline HCPs during the current COVID-19 pandemic. Therefore, the purpose of this study was to gain an in-depth understanding of frontline healthcare professionals’ experiences of balancing work life and family life during the COVID-19 pandemic.

## Methods

This study applied a qualitative explorative design using individual interviews. Ricoeur´s phenomenological hermeneutical philosophy created the epistemological stance for exploring HCPs’ lived experiences of how they balanced work life and family life during the current COVID-19 pandemic. This approach offers a frame in which participants’ lived experiences can be interpreted and thus, a comprehensive understanding can be achieved.

### Sample

Participants in this study were recruited from a population of HCPs who had a frontline caring and/or treatment responsibility for patients infected with COVID-19 during the first phase of the pandemic in Denmark. These HCPs were not normally necessarily employed to care for or treat infectious patients specifically but became part of the emergency response at the onset of the pandemic due to either their specific competencies or on a voluntary basis. Specific competencies, in addition to being a specialist in infectious diseases, could involve intensive, anesthesiologic, cardiopulmonary or other relevant care and treatment experience and knowledge, while HCPs from other medical areas signed up at a voluntary basis due to the urgency of the situation. We used a convenience sampling strategy [20] by encouraging HCPs from different regions of Denmark to approach the research team by phone or e-mail if they were willing to attend an interview. The interviews were conducted by telephone based on ethical accountability for not contributing to the spread of the virus. Twenty-two HCPs consented to participate in interviews from May to August 2020. Data saturation was thereafter achieved, making further interviewing unnecessary [20]. HCPs with different professional backgrounds and different responsibilities were included. The characteristics of the participants are shown in Table 1.

**Table 1:** Sociodemographic characteristics of the included participants

Descriptions	Number (n)
**Region in Denmark**	
Capital	14
Zealand	2
North Jutland	2
Central Jutland	4
Southern Denmark	0
Sex	
Female	12
Male	10
**Age**	
21–30	6
31–40	9
41–50	4
51–70	3
**Profession**	
Nurse	8
Doctor	6
Physiotherapist	5
Medical Laboratory Technologist	1
Occupational therapist	1
Porter	1
**Years in profession**	
0–10 years	9
11–20 years	10
21–44 years	3
**Original Specialty / Department**	
Intensive care	8
Accident and emergency	2
Infectious disease	1
Pediatrics	1
PhD student / Researcher	3
Gastrointestinal	1
Ear, nose and throat (ENT)	1
Pulmonology	2
Cardiology	1
Endocrinology	1

### Data collection

According to Ricoeur, human experiences are characterized by unreflecting preunderstanding [21, 22]. In order to gain access to the lived experiences of caring for, and treating patients with COVID-19, a narrative approach was used for data collection during individual interviews. The participating HCPs’ expressions reflected their experiences as they saw them and wanted to present them. The interviews were open-ended in order to explore the HCPs’ lived experiences and emphasised listening to them. Narrative accounts of participant experiences were encouraged but questions such as “Could you please tell me about how you experience caring for patients with COVID-19?” and “How do you feel about being on the frontline at work during such a pandemic while also being part of a family with accompanying responsibilities?” were used during the interviews. Table 2 lists the interview questions. Four experienced qualitative researchers, who were not a part of the clinical care and treatment for COVID-19 infected patients, performed the interviews. The researchers interviewed HCP’s that they did not know in advance in order to be as open and curious as possible. The interviews lasted on average 25 min and were recorded and transcribed.

**Table 2:** Interview questions

Could you please tell about:
• What thoughts you had before you embarked on the task/job?
• What it means for you to care for / treat patients with COVID-19 - both professionally and personally?
• How you have experienced the collaboration with colleagues during the period?
• What thoughts you have had about infection? (at work/at home/in society)

### Ethical considerations

 The study was approved by the Danish Data Protection Agency (P-2020-276) and was undertaken in accordance with the guidelines of the Danish Research Ethics Review of Health Research Projects. The principles outlined in the Declaration of Helsinki was followed. Written information about the purpose of the study and the right to withdraw at any time was handed out to the participating HCPs before deciding to participate. Written informed consent was obtained from each of the participants before the interview. Data were anonymized by means of identification codes and the participating HCPs were informed that interview data would be treated confidentially.

### Data analysis

Embedded in language, according to Ricoeur [21, 22], is always a meaning that extends beyond the direct linguistic expression and such linguistic connotations can only be approached through a process of interpretation. A Ricoeur-inspired interpretation process consists of different layers of meaning understood as an endless spiral involving three levels: a naive search for the overarching meaning which the text seeks to convey, a linguistically oriented structural analysis, and an in-depth comprehensive interpretation [22, 23]. First, reading and re-reading of the transcribed texts took place to gain an initial understanding of what the texts were about as a naive interpretation of the narratives. Secondly, a structural analysis was performed providing an insight into the structure of the texts. Words and sentences which pointed towards an issue or a theme that recurred throughout the texts were extracted. The structural analysis exceeded the naive understanding and enabled a more in-depth interpretation. At the third level of analysis we continued with an in-depth interpretation and discussion of the themes identified in the structural analysis. Where a structural analysis solely was aimed at a closed system of symbols, this comprehensive interpretation aimed at understanding the meaning of frontline HCPs’ experiences of balancing work life and family life during the COVID-19 pandemic. All authors performed the analysis and interpretation.

## Results

The understanding of frontline HCPs’ experiences of balancing work life and family life during the COVID-19 pandemic are described in three themes: (1) Readiness for change, (2) A sense of being abandoned among family, and (3) Opposing feelings about being a part of something bigger. In the following, these themes will be illuminated in-depth one by one complemented by sub-themes, while Table 3 is showing the key areas to enhance the visibility to the reader.

**Table 3:** Key areas of frontline healthcare professionals’ experiences of balancing work life and family life during the COVID-19 pandemic

Readiness for change	Frontline healthcare professionals treating and caring for patients with COVID-19 are, voluntarily or involuntarily, forced to be ready to change departments as well as being ready to face the unknown coronavirus.
**A sense of being abandoned among family**	The frontline work leads to feelings of being abandoned among their families and friends due to the threat of bringing the infection home and spreading the virus.
**Opposing feelings about being a part of something bigger**	Although healthcare professionals are facing a working life filled with uncertainty and unpredictability impacting their family life, they express opposing feelings of being a part of something bigger.

### Readiness for change

#### Feelings of being pressured

The participating HCPs described how they were forced by the acute outbreak to be ready for changes. A sense of having a say as well as having the opportunity of choosing to work in COVID-19 wards was crucial for their readiness for change and motivation to work at short notice. While some HCPs found it natural to be part of the frontline, others felt pressured to do so and described feelings of being deprived of the right to choose whether they wanted to work in a specialized COVID-19 ward or not.

*I have not signed up to work in the COVID ward…. She [the boss] called and said that she had been instructed to find three [HCPs for the COVID-19 ward], and she had half an hour to do that. She was a little panicked and I could hear the hidden: “we are counting on you” in her voice. She also said that two others already had agreed to the task, so they just needed me.*

This involuntary pressure experienced by the HCPs pushed them to be ready for changes. For some HCPs this push meant they were growing and felt important and ready for the changes while for others it had a negative impact on their professional identity and job satisfaction. Not only a readiness for changing working environments and physical sites were expressed by the HCPs but also a readiness for facing a novel and unknown coronavirus was dominating their narratives. They described how they had to be ready for something they did not know about and as such did not know how to prepare for. To be ready for something unknown was approached with a certain variation in motivation from the HCPs. An important factor for the individual’s motivation and readiness to face the more or less unknown working conditions was highlighted as being involved in decisions about expected tasks and responsibilities. Furthermore, perceptions of being heard and invited to reflect on possible consequences of agreeing to be caring for and treating patients with COVID-19 infection was expressed as paramount for the participating HCPs’ readiness for changes.

*I received an email from my manager late at night, where she asked if I wanted to join*.

*[the COVID contingency], but I did not see it [the e-mail]. So, when I came to work the next morning, she [the manager] was ready before I even met and asked if she could count on me.*

#### A threat to health for the HCPs and family

The consequences of not being able to reflect and discuss possible interventions and potential fears related to one’s own family life filled the HCPs with mixed feelings of, on the one hand, an obligation to be ready while it, on the other hand, was difficult to see and think through the possible interferences in one’s own life and family. A prominent described threat to the HCPs’ readiness for changes was the potential threat to their own as well as their family’s health. The participating HCPs expressed their experiences of a previously unknown uncertainty in how to balance their working obligations and readiness to be on the front in the care and treatment of patients with COVID-19 and their family life. Suddenly they feared for their own health but also extensive concerns about bringing COVID-19 home to their families was illuminated. This fear was for some of the HCPs faced and placed in the background of their consciousness; while others carried the fear with them as a burden, influencing their readiness. These HCPs did, however, try to divert the fear and do their job: *“I try not to stress over things that I can’t influence. If it comes [being infected and bringing illness home], then I will do my best”.*

#### The professional pride

From being an expert in one’s own field to a novice in a new department was associated with uncertainties with having to balance a new way of being a professional. Despite uncertainty about caring for and treating patients with COVID-19, cohesion among the HCPs was highlighted. They described how every HCP contributed with their knowledge and skills leading to a sense of professional pride and common goals and direction. A strong metaphor of being in a war together and stepping in when your help was needed was pervasive in the HCPs’ narratives: *”It was like being at war … You could say that we were the soldiers that were going to be in the front*”. This readiness for stepping in did, however, have consequences and required changes and flexibility at home. Changes in working hours and variety of shifts had a substantial impact on family life and, thereby, the work life balance. Despite these challenges in balancing working life and family life, experiences of this very special team spirit providing an extraordinary effort in the fight against COVID-19 were pointed out as being worth it. Although it was mentally exhausting being surrounded by colleagues you did not know in advance, it was also experienced as a great strength to work with colleagues from different specialties with a mutual goal of making an effort of helping out in a situation associated with multiple challenges for the involved individuals: *“This has been the best thing I have experienced in my career. Seeing nurses who stopped working three, five and ten years ago come in and say: I want to help*”. This perceived community and the experience that everyone contributed with each their professionalism and enthusiasm was of great importance for the individual HCPs’ readiness for change: *“The best thing about being part of this process was those people…, they ignored the obstacles that there usually are… It was people that just made things happen”.*

### A sense of being abandoned among family

#### The poisonous HCP

HCPs working frontline taking care of and treating patients with COVID-19 described ambiguous experiences of being abandoned among family and friends due to the risk of infection through their work. The very real possibility of the HCPs becoming infected and catching COVID-19 due to close contact with infected patients raised concerns from their families who began regarding them as potentially poisonous from whom one should distance themselves.

*He [son] didn’t want to be near me; not only because he himself could be infected, but he was also afraid of infecting others. He wished that his mother could be without it [the frontline care and treatment for COVID-19 patients]*.

Experiences of family members keeping extra distance thus permeated the stories of the HCPs and such experiences became ordinary daily events when meeting others. In the HCPs’ daily life and surroundings, they felt isolated from usual social contact with close family and descriptions of relatives moving out of the shared home in fear of being infected were illuminated.

*My sister, her husband and my two-year-old niece said they were isolating themselves from me. My boyfriend lived with others and said I should not visit him there while working so closely with corona patients. There were several who withdrew from me [in social surroundings].*

#### A high price to pay

The consequences for the family life of HCPs working forefront in the COVID-19 departments thus led to feelings of being abandoned. Further consequences concerning the children of the HCPs were also described. All of a sudden, their children did not get invited to playdates and some of their usual friends were not allowed to play with children of HCPs. The social consequences for the HCPs’ children was expressed as a very high price to pay in order to meet their obligations of caring for and treating patients with COVID-19 and experiences of stigma was also expressed in the stories.

The HCPs did, however, also take precautions and distanced themselves from their family where possible: *“I was not nervous for myself [as a HCP] getting infected, but for me to infect my family. That is why we [HCP and spouse] kept our distance from our family. We were completely isolated; we were only being with ourselves”.* This contrast between being abandoned by one’s family and the voluntary distancing and isolation from friends and family was expressed as an important factor in the work life and family life balance. This balance could easily be disturbed if feelings of being abandoned by family and friends dominated, leading to a sense of doubt about if the frontline work and obligations were worth it. However, the voluntary distance to their family contributed to the HCPs’ working identity and helped maintain the balance between work and family in a way that felt trustworthy for themselves.

#### The stigmatized HCP

Although the feeling of being distanced from the family could either be characterized by fear and stigma from the social environment, the HCPs understood very well this distance. Uncertainties about the novel coronavirus contributed to awareness among the HCPs about selecting when and with whom to interact socially. Likewise, the participating HCPs experienced a great deal of concern from their loved ones due to their potential risk of getting infected with COVID-19 combined with respect towards them due to working in the forefront in the care and treatment of patients with COVID-19 and thereby risking their own safety.

### Opposing feelings about being a part of something bigger

#### A fighting spirit

The HCPs described how they experienced a tension marked by opposing feelings that could be difficult to deal with. On the one hand, they talked about how they wanted to be part of the fight against COVID-19 and make a difference for the sick patients in the worldwide pandemic, while at the same time experiencing a work life full of insecurity. Their stories illuminated how thinking about being a part of something bigger contributed to a fighting spirit and professional pride. These feelings did, however, for some HCPs, fade away when actually working in the department caring for and treating the COVID-19 patients.

In contrast to the imagined expectations of being part of a bigger contingency - namely a team of professionals fighting an unknown virus - insecurity and ambivalent feelings of not wanting to quit their usual jobs and letting something unfamiliar and unknown impact their family life was illuminated in the HCPs’ narratives.

*It was frustrating to quit my regular job and then suddenly have to do something completely different. At the same time, it was an extraordinary situation that no one had tried before. So of course, I also felt that I had to step in and help. But I had an ambivalent feeling about it. Basically, I would probably have preferred not to be a part of it.*

#### Putting own work on hold

An overwhelming sense of frustration due to putting their own work on hold with an uncertain time horizon without knowing when to resume was described as particularly important for the HCPs’ adaptability of working frontline caring and treating for patients with COVID-19. The fact that the participating HCPs did not know when to resume their regular job had an impact on their motivation for doing their work and participating in the COVID-19 contingency. The HCPs also described how conditions such as working hours and shifts as well as tasks constantly changed leading to a sense of unpredictability and exhaustion. Tasks that exceeded one’s competencies proved especially discouraging. Such challenges were difficult to deal with and the HCPs had an urgent need for sharing these with someone. When the HCPs experienced being listened to by a colleague or their closest manager, they described how they felt a relief. This relief was expressed if the listening led to a balance between what expectations were placed on them and the skills they had, but also when the flexibility that the individual HCP had in relation to their family life was recognized. So, when sharing frustrations and uncertainties about working life, a balance with family life could be maintained for the HCPs, however, experiences of being left to oneself had a negative impact on their family life in which the HCPs had to place their frustrations.

#### Being part of a professional set-up

Despite the possible overwhelming experience of being on the front in a pandemic, the HCPs also described how being part of a professional setup that really worked and where everyone took an active part and were responsible, dedicated and motivated gave a feeling of being part of something bigger. The participating HCPs related how everyone helped where they could and did their very best and how being a part of this big picture gave a sense of professional pride: *“As a nurse, I should not sit in my PhD office. You became a nurse, because you want to help when there is a need, and there was a need, and therefore I liked to join”.* This relief was about balancing the expectations they had and the skills they had, but also the flexibility that the individual had in relation to their family life. A sense of agreement among the HCPs was illuminated in their narratives and they described how they experienced almost never being denied help from colleagues and how everybody focused on helping each other. This helping community was particularly significant for performing in this extraordinary situation. Furthermore, this community and feelings of being part of something bigger led to a special vigilance among the HCPs and their well-being which was important for balancing work life and family life. Feelings of being part of something bigger thus outweighed the possible negative consequences for the family life.

#### A shadow of fear

 Although the HCPs described the importance of being part of something bigger, they also talked about opposing feelings on the way to their first day of work when they had to meet, care for and treat their first patient with COVID-19. Their stories illuminated how their motivation to contribute with their knowledge and skills could be overshadowed by fear of the uncertain and unknown, and how the risk of getting infected themselves or infecting their family and friends were in the foreground of their consciousness in the beginning.

*Before the first shift I was sitting in the car wondering if I was scared, even though I knew the isolation regimen. We [as HCPs] end up getting infected ourselves… So, I had such a thought on the way into [the hospital] about what this [COVID-19] was, for nobody knew what it was and how dangerous it really was.*

These thoughts of how dangerous the disease might be was most prevalent in the beginning of the individual HCPs’ meeting with infected patients, however, such thoughts continued to lie as a shadow both in the HCPs’ working life as well as in their family life witnessing the seriousness of the situation.

## Discussion

This study is one of only a few studies offering an in-depth understanding of frontline HCPs’ experiences of balancing work life and family life during the COVID-19 pandemic. A prominent disturbing factor for the participating HCPs’ family life was the fact that they, with the outbreak of the COVID-19 pandemic, all of a sudden had to be ready for change. They had to be ready for not only changing departments, working hours and shifts - but also a readiness to face an unknown virus. This readiness for change caused feelings of an involuntary pressure of being forced into a new and unknown working situation and responsibilities. A serious threat to work life balance for the HCPs in the present study was the risk of bringing infection home to the family, which is highlighted as more frightening than the threat to one’s own health. Such reactions during infectious disease outbreaks has been reported elsewhere [10, 24], and these behavioral responses are designed to counteract fear during a pandemic and may attenuate the threat to one’s own health [25]. Balancing work life sufficiently fosters not only satisfaction in one’s job, performance and organizational commitment but feelings of caring for the family is paramount [26, 27], which, however, was challenged for the HCPs in the present study.

For some HCPs being ready for change and stepping up to treat and care for patients with COVID-19 leads to a sense of personal and professional growth while others described a negative impact on their professional identity. Evidence in occupational health literature shows that employee control over work performance is linked with health outcomes, and not experiencing this control might lead to exhaustion and depressive symptoms, impact on physical and mental health as well as work family conflicts [28–30]. Norms around work time are also about space; about being at the workplace during certain hours which structures the lives of individuals in profound ways [28]. This structure was, however, interrupted for the participants in this study. Despite descriptions of disrupted temporal structures regarding work schedules and work hours challenging the work life balance, a metaphor of being in war together highlighted cohesion and team spirit among HCPs. It has been reported that HCPs, despite experiencing strong pressure of fear of infection, exhaustion by heavy workloads and stress of caring for seriously ill COVID-19 patients still present a strong sense of duty and identity as a HCP [15, 31]. This dedication towards their work should, however, not overshadow and create imbalance regarding a HCPs’ family life [27, 32]. Working for a healthcare system that is prepared and has an effective plan is magnified many times over in a pandemic and should be prioritized. It has further been reported how offering free shuttle services between work and home, childcare support and meal vouchers for staff may reduce domestic stress and allow for a single-minded effort for HCPs [33].

In the present study the HCPs described a sense of being abandoned among their family and friends due to their work on the frontline of treating and caring for patients with COVID-19. Such experiences of being abandoned made the HCPs feel isolated from usual social contact. Research has described examples of how e.g. nurses working forefront in caring for patients with COVID-19 chose to hide the fact from their family and friends that they were working at a COVID-19 department in fear of how their family would react [10]. The consequence of being abandoned points at a disturbance in the work life balance which may cause psychological distress for the HCPs [28]. In general high work demands have been shown to be related to an increased degree of conflict between work life and family life [34, 35], however, this study highlights a new perspective of interest; namely a conflict based on fear. This fear creates a distance between HCPs and their families leading to a conflict within the individual HCP if their work on the frontline is worth it or if it is too high of a price to pay. Research has pointed at such ethical challenges during previous disaster outbreaks where the HCPs’ personal and possibly their family’s lives and health are at risk, and they must weigh the option of continuing to work or retreat to safety [36]. Such decisions that are made daily, are based on professional and personal values of how they perceive existing risks. It is further described how HCPs, during uncertain times, e.g. the current COVID-19 pandemic, are looking for trust, compassion, stability and hope from their leaders [37]. They want to be part of the work solutions and are incredibly resilient when action plans are clear which helps control mental health issues raised by an imbalance between work life and family life [38, 39] such as the described deselection of the participating HCPs. In this study it was further described how HCPs who themselves voluntarily chose to keep family and friends at a distance, could more easily maintain a meaningful work life balance.

For the participating HCPs in this study, opposing feelings of being a part of something bigger was expressed. On the one hand their working life on the frontline COVID-19 contingency was shadowed with uncertainty and unpredictability and they described how these new and unknown working conditions impacted on their usual family life. On the other hand, however, these feelings of being part of something bigger contributed to a fighting spirit and professional pride which outweighed the negative consequences; like being soldiers on the front. Research has reported how HCPs might grow personally and professionally under pressure from being on the frontline caring and treating for COVID-19 patients and during other pandemics [10, 40]. A work life balance is among other factors depending on a self-perceived professional job satisfaction [41]. Moreover, a congruence in work family integration indicates that an individual’s own resource allocation decisions toward work and family are validated by their organization [42]. Leadership during the COVID-19 pandemic is as such paramount in supporting the frontline HCPs in being part of a community and an environment in which helping each other is in focus in the fight against the coronavirus. Factors that guide HCPs to respond are very personal and highly connected to balancing work life and family life, however, it is reported how organizational and professional leadership can modify those factors to increase the number that are willing to put the needs of COVID-19 patients first [36, 43].

### Limitations

Through a qualitative approach, we were able to explore the experiences of frontline HCPs in balancing work life and family life during the COVID-19 pandemic. It is worth noticing that the thoughts and experiences presented in this study are the most dominant among the included HCPs, however, such dominant ideas may differ among age groups. Due to the small sample size, we have not been able to differentiate according to an age perspective which otherwise would have been particularly interesting in the light of balancing a work life and family life; some being young and striving for a career, or being a family with young kids, or maybe being a senior with a potential threat to own health while caring for COVID-19 patients. Another important opt out perspective are the described experiences of feeling pressured as a HCP caring for COVID-19 patients while some HCPs did volunteer for the task. This issue could be significant to address in a further study. Despite the disadvantages of telephone interviews reducing social cues [44], as this type of interview does not allow body language to be used as a source of additional information, we found that the subjects were willing to participate in the study and appreciated sharing their experiences. Regarding the applied analysis and interpretation, it is important to note that there is always more than one way to interpret a text. The interpreted comprehensive understanding in this article is what we found most probable from what the participants told in the narratives based on the researchers’ preunderstandings [22]. We furthermore used a convenience sampling strategy, as it was a rapid way to address frontline HCPs’ experiences of treating and caring for patients with COVID-19, and thus a relatively efficient method for gathering data in a situation where such data is highly relevant and urgently needed. The disadvantages of this sampling method are, however, that it may include people with resources and mental surplus [20], which the reader should consider when transferring the findings to other settings.

## Conclusions

Healthcare systems worldwide are being put to the ultimate test and are under tremendous pressure to limit the spread of the novel coronavirus. Most of this responsibility is being shouldered by frontline HCPs. In the present study we describe how HCPs are forced to be ready to change departments and be those frontline individuals, voluntary as well as involuntary, treating and caring for patients with COVID-19 infection as well as being ready to face the unknown coronavirus. The work life balance for these HCPs are threatened by changes in professional responsibilities and working hours and shifts. Furthermore, the frontline HCPs experience being abandoned among their families and usual social circles due to the threat of bringing the infection home and spreading the virus. This fear challenges them ethically and create a distance between HCPs and their families leading to a conflict within the individual HCP if their work on the frontline is worth it or if it is a too high price to pay. Although an unknown and unfamiliar coronavirus impacts HCPs’ family life, the participating HCP expressed opposing feelings of being a part of something bigger. They faced a working life filled with uncertainty and unpredictability, but at the same time felt being a part of something bigger that contributed to a fighting spirit and professional pride outweighing the negative consequences; like being soldiers on the front.

### Clinical implications

In a clinical perspective these new findings can be used to guide management during the pandemic. It seems that these working conditions are not sustainable over a long period of time due to the consistent psychological and physical threat of being infected, infecting the family and working in warm and uncomfortable equipment causing physical symptoms. This calls for flexibility in work schedule and periods where risk of contamination of family is low, so that the HCPs can be with their family in a safe manner. All management, organizational and political tools must be used to make it attractive for HCPs to volunteer to work frontline, e.g. sufficient and qualified staffing in each shift, access to appropriate protective equipment, salary reflecting the importance and risk of the task and individually planed schedules.

## Data Availability

All authors have full control of all primary raw data (interview transcripts) and allow the journal to review our data if requested. All raw data are written in Danish. Data are stored in a locked file cabinet in a locked room at the Copenhagen University Hospital as requested by the Danish Data Protection Agency. The data material used in this study are available from the corresponding author on reasonable request which will not conflict with the anonymity and confidentiality of the data.

## Abbreviations

HCP: Healthcare professionals

## References

[1] World Health Organization. Novel Coronavirus (2019-nCoV), situation report-3 [Internet]. 2020 Jan [cited 2020 Dec 14]. Available from: https://apps.who.int/iris/bitstream/handle/10665/330762/nCoVsitrep23Jan2020-eng.pdf

[2] Pascarella G, Strumia A, Piliego C, Bruno F, Del Buono R, Costa F, et al. COVID-19 diagnosis and management: a comprehensive review. J Intern Med. 2020 Aug;288(2):192–206. 10.1111/joim.13091 PMC7267177 32348588

[3] Rothan HA, Byrareddy SN. The epidemiology and pathogenesis of coronavirus disease (COVID-19) outbreak. 2020 May;109:102433. 10.1016/j.jaut.2020.102433 PMC7127067 32113704

[4] Jin YH, Cai L, Cheng ZS, Cheng H, Deng T, Fan YP, et al. A rapid advice guideline for the diagnosis and treatment of 2019 novel coronavirus (2019-nCoV) infected pneumonia (standard version). Vol. 7, Military Medical Research. BioMed Central Ltd.; 2020 Feb 6;7(1):4. 10.1186/s40779-020-0233-6 PMC7003341 32029004

[5] Barranco R, Ventura F. Covid-19 and infection in health-care workers: An emerging problem. Med Leg J. 2020 Jul 1;88(2):65–6. 10.1177/0025817220923694 32441196

[6] Demartini K, Konzen V de M, Siqueira M de O, Garcia G, Jorge MSG, Batista JS, et al. Care for frontline health care workers in times of covid-19. Rev Soc Bras Med Trop. 2020;53:e20200358. 10.1590/0037-8682-0358-2020 PMC7451495 32876318

[7] Neto MLR, Almeida HG, Esmeraldo JD ar., Nobre CB, Pinheiro WR, de Oliveira CRT, et al. When health professionals look death in the eye: the mental health of professionals who deal daily with the 2019 coronavirus outbreak. Vol. 288, Psychiatry Research. 2020 Jun;288:112972. 10.1016/j.psychres.2020.112972 PMC7152886 32302817

[8] Gómez-Ochoa SA, Franco OH, Rojas LZ, Raguindin PF, Roa-Díaz ZM, Wyssmann BM, et al. COVID-19 in Health-Care Workers: A Living Systematic Review and Meta-Analysis of Prevalence, Risk Factors, Clinical Characteristics, and Outcomes. Am J Epidemiol. 2020 Sep 1;kwaa191. 10.1093/aje/kwaa191 PMC7499478 32870978

[9] Pappa S, Ntella V, Giannakas T, Giannakoulis VG, Papoutsi E, Katsaounou P. Prevalence of depression, anxiety, and insomnia among healthcare workers during the COVID-19 pandemic: A systematic review and meta-analysis. Vol. 88, Brain, Behavior, and Immunity. 2020 Aug;88:901–907. 10.1016/j.bbi.2020.05.026 PMC7206431 32437915

[10] Sun N, Wei L, Shi S, Jiao D, Song R, Ma L, et al. A qualitative study on the psychological experience of caregivers of COVID-19 patients. Am J Infect Control. 2020 Jun;48(6):592–598. 10.1016/j.ajic.2020.03.018 PMC7141468 32334904

[11] Xiang YT, Yang Y, Li W, Zhang L, Zhang Q, Cheung T, et al. Timely mental health care for the 2019 novel coronavirus outbreak is urgently needed. Vol. 7, The Lancet Psychiatry. 2020 Mar;7(3):228–229. 10.1016/S2215-0366(20)30046-8 PMC7128153 32032543

[12] Smith GD, Li WHC (2020). COVID-19: Emerging compassion, courage and resilience in the face of misinformation and adversity. J Clin Nurs.

[13] Kitson AL. The Fundamentals of Care Framework as a Point-of-Care Nursing Theory. Nurs Res. 2018 Mar 1;67(2):99–107. 10.1097/NNR.0000000000000271 29489631

[14] Hillier MD. Using effective hand hygiene practice to prevent and control infection. Nurs Stand. 2020 Apr 29;35(5):45–50. 10.7748/ns.2020.e11552 32337862

[15] Missel M, Bernild C, Dagyaran I, Christensen SW, Berg SK. A stoic and altruistic orientation towards their work: a qualitative study of healthcare professionals’ experiences of awaiting a COVID-19 test result. BMC Health Serv Res. 2020 Nov 11;20(1):1031. 10.1186/s12913-020-05904-0 PMC7656225 33176771

[16] Drexler R, Hambrecht JM, Oldhafer KJ. Involvement of Medical Students During the Coronavirus Disease 2019 Pandemic: A Cross-Sectional Survey Study. 2020 Aug 30;12(8):e10147. 10.7759/cureus.10147 PMC7526758 33014645

[17] Sethi BA, Sethi A, Ali S, Aamir HS. Impact of coronavirus disease (COVID-19) pandemic on health professionals. Pakistan J Med Sci. 2020 May;36(COVID19-S4):S6-S11. 10.12669/pjms.36.COVID19-S4.2779 PMC7306959 32582306

[18] Hjálmsdóttir A, Bjarnadóttir VS. “I have turned into a foreman here at home”: Families and work–life balance in times of COVID-19 in a gender equality paradise. Gender, Work Organ. 2020 Sep 19;10.1111/gwao.12552. 10.1111/gwao.12552 PMC7537149 33041540

[19] Haar JM, Russo M, Suñe A, Ollier-Malaterre A. Outcomes of work-life balance on job satisfaction, life satisfaction and mental health: A study across seven cultures. J Vocat Behav. 2014;85(3).

[20] Polit DF, Beck CT. Nursing research: generating and assessing evidence for nursing practice. 11th ed. Wolters Kluwer; 2020.

[21] Missel M, Birkelund R. Ricoeur’s narrative philosophy: A source of inspiration in critical hermeneutic health research. Nurs Philos. 2019 May 14;e12254. 10.1111/nup.12254 31087495

[22] Ricoeur P (1976). Interpretation Theory. Discource and the Surplus of Meaning.

[23] Simonÿ C, Specht K, Andersen IC, Johansen KK, Nielsen C, Agerskov H. A Ricoeur-Inspired Approach to Interpret Participant Observations and Interviews. Glob Qual Nurs Res. 2018 Oct 30;5:2333393618807395. 10.1177/2333393618807395 PMC6207962 30397637

[24] Lee SH, Juang YY, Su YJ, Lee HL, Lin YH, Chao CC, Facing SARS (2005). Psychological impacts on SARS team nurses and psychiatric services in a Taiwan general hospital. Gen Hosp Psychiatry. Sep-Oct.

[25] Shultz JM, Cooper JL, Baingana F, Oquendo MA, Espinel Z, Althouse BM, et al. The Role of Fear-Related Behaviors in the 2013–2016 West Africa Ebola Virus Disease Outbreak. Current Psychiatry Reports. 2016 Nov;18(11):104. 10.1007/s11920-016-0741-y PMC5241909 27739026

[26] Gragnano A, Simbula S, Miglioretti M. Work–life balance: weighing the importance of work–family and work–health balance. Int J Environ Res Public Health. 2020 Feb 1;17(3):907. 10.3390/ijerph17030907 PMC7037206 32024155

[27] Sirgy MJ, Lee DJ (2018). Work-Life Balance: an Integrative Review. Applied Research in Quality of Life.

[28] Moen P, Kelly EL, Tranby E, Huang Q. Changing work, changing health: Can real work-time flexibility promote health behaviors and well-being? J Health Soc Behav. 2011 Dec;52(4):404–29. 10.1177/0022146511418979 PMC3267478 22144731

[29] Stansfeld S, Candy B. Psychosocial work environment and mental health - A meta-analytic review. Scand J Work Environ Heal. 2006 Dec;32(6):443–62. 10.5271/sjweh.1050 17173201

[30] De Lange AH, Taris TW, Kompier MAJ, Houtman ILD, Bongers PM. The relationships between work characteristics and mental health: Examining normal, reversed and reciprocal relationships in a 4-wave study. Vol. 18, Work and Stress. 2004.

[31] Liu YE, Zhai ZC, Han YH, Liu YL, Liu FP, Hu DY. Experiences of front-line nurses combating coronavirus disease-2019 in China: A qualitative analysis. Public Health Nurs. 2020 Sep;37(5):757–763. 10.1111/phn.12768 PMC7405388 32677072

[32] Jackson D, Firtko A, Edenborough M. Personal resilience as a strategy for surviving and thriving in the face of workplace adversity: A literature review. J Adv Nurs. 2007 Oct;60(1):1–9. 10.1111/j.1365-2648.2007.04412.x 17824934

[33] Nagesh S, Chakraborty S. Saving the frontline health workforce amidst the COVID-19 crisis: Challenges and recommendations. Vol. 10, Journal of Global Health. 2020 Jun;10(1):010345 10.7189/jogh-10-010345 PMC7183244 32373323

[34] Albertsen K, Kristensen TS, Pejtersen J. Lange og skæve arbejdstider — kan øget indflydelse bedre balancen? [Long and skewed working hours - can increased influence improve balance?] Tidsskr Arb. 2007 Mar 1;9(1):61.

[35] Bajaj AK. Work/Life Balance: It Is Just Plain Hard. Vol. 80, Annals of plastic surgery. 2018 May;80(5S Suppl 5):S245-S246. 10.1097/SAP.0000000000001415 29596086

[36] Iserson K V. Healthcare ethics during a pandemic. Vol. 21, Western Journal of Emergency Medicine. 2020 Apr 13;21(3):477–483. 10.5811/westjem.2020.4.47549 PMC7234717 32302284

[37] Phillips JA. Work–Life Fit During A Pandemic. Vol. 68, Workplace Health and Safety. SAGE Publications Inc.; 2020. p. 502–3. 10.1177/2165079920953830 32924888

[38] Shanafelt T, Ripp J, Trockel M. Understanding and Addressing Sources of Anxiety among Health Care Professionals during the COVID-19 Pandemic. JAMA - J Am Med Assoc. 2020 Jun 2;323(21):2133–4. 10.1001/jama.2020.5893 32259193

[39] Blake H, Bermingham F, Johnson G, Tabner A. Mitigating the psychological impact of covid-19 on healthcare workers: A digital learning package. Int J Environ Res Public Health. 2020 Apr 26;17(9):2997. 10.3390/ijerph17092997 PMC7246821 32357424

[40] Kang HS, Son YD, Chae SM, Corte C. Working experiences of nurses during the Middle East respiratory syndrome outbreak. Int J Nurs Pract. 2018 Oct;24(5):e12664. 10.1111/ijn.12664 PMC7165521 29851209

[41] Liu P, Wang X, Li A, Zhou L. Predicting Work–Family Balance: A New Perspective on Person–Environment Fit. Front Psychol. 2019 Aug 6;10:1804. 10.3389/fpsyg.2019.01804 PMC6691148 31447741

[42] Grawitch MJ, Maloney PW, Barber LK, Mooshegian SE. Examining the nomological network of satisfaction with work-Life balance. J Occup Health Psychol. 2013 Jul;18(3):276–84. 10.1037/a0032754 23688250

[43] Iserson K V., Heine CE, Larkin GL, Moskop JC, Baruch J, Aswegan AL. Fight or Flight: The Ethics of Emergency Physician Disaster Response. Ann Emerg Med. 2008 Apr;51(4):345–53. 10.1016/j.annemergmed.2007.07.024 PMC7124291 17950487

[44] Davies L, LeClair KL, Bagley P, Blunt H, Hinton L, Ryan S, et al. Face-to-Face Compared With Online Collected Accounts of Health and Illness Experiences: A Scoping Review. Qual Health Res. 2020 Nov 1;30(13):2092–102. 10.1177/1049732320935835 32667257

